# Supplementary figures and images for: HOX Gene Expressions in Cultured Articular and Nasal Equine Chondrocytes
Source: Animals (Basel). 2021 Aug 30;11(9):2542. doi: 10.3390/ani11092542 (PMC8471089; doi:10.3390/ani11092542)

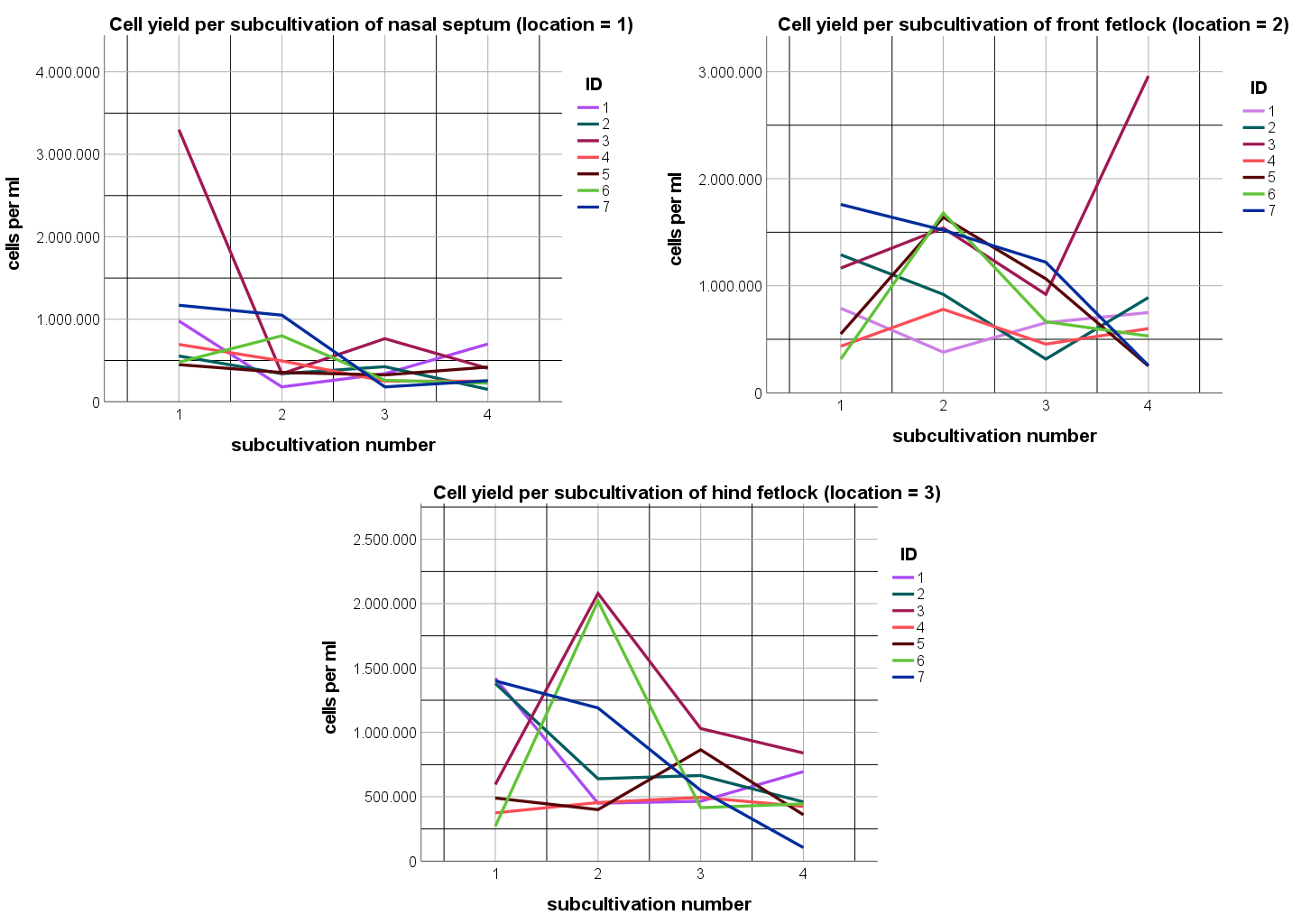

Supplement: Supplementary file 1 [file animals-11-02542-s001.zip › Supplementary Figure S1_ cell_yields.png]
